# Prevalence of Psychiatric Disorders among Children and Adolescents in Northeast China

**DOI:** 10.1371/journal.pone.0111223

**Published:** 2014-10-31

**Authors:** Yang Xiaoli, Jiang Chao, Pan Wen, Xu Wenming, Liang Fang, Li Ning, Mu Huijuan, Na Jun, Lv Ming, An Xiaoxia, Yu Chuanyou, Fu Zenguo, Li Lili, Yu Lianzheng, Tong Lijuan, Pan Guowei

**Affiliations:** 1 Institute of Chronic Disease, Liaoning Provincial Center for Disease Control and Prevention, Shenyang city, China; 2 Department of Psychiatry, Dalian Medical University, Dalian city, China; 3 Department of Chronic Disease, Panjin Oil Field Center for Disease Control and Prevention, Panjin city, China; 4 Department of Psychiatry, Dalian Municipal Hospital of Psychiatric Disease, Dalian city, China; 5 Department of Chronic Disease, Donggang County Municipal Center for Disease Control and Prevention, Donggang county, Liaoning province, China; 6 Department of Chronic Disease, Benxi Municipal Center for Disease Control and Prevention, Benxi, China; 7 Department of Chronic Disease, Qingyuan County Center for Disease Control and Prevention, Qingyuan county, Liaoning province, Qingyuan, China; 8 Department of Chronic Disease, Zhangwu County Center for Disease Control and Prevention, Zhangwu county, Liaoning province, Zhangwu, China; 9 Department of Psychiatry, the 1st Attached Hospital of China Medical University, Shenyang city, China; Department of Chronic Disease, Panjin Municipal Center for Disease Control and Prevention, Panjin city, China; Hamamatsu University School of Medicine, Japan

## Abstract

**Background:**

To describe the prevalence of DSM-IV disorders and comorbidity in a large school-based sample of 6–17 year old children and adolescents in northeast China.

**Methods:**

A two-phase cross-sectional study was conducted on 9,806 children. During the screening phase, 8848 children (90.23%) and their mothers and teachers were interviewed using the Strengths and Difficulties Questionnaire (SDQ). During the diagnostic phase, 1129 children with a positive SDQ and 804 randomly selected children with a negative SDQ (11%), and their mothers and teachers, were interviewed using the Development and Well-Being Assessment (DAWBA).

**Results:**

The overall prevalence of DSM-IV disorders was 9.49% (95% CI = 8.10–11.10%). Anxiety disorders were the most common (6.06%, 95% CI = 4.92–7.40), followed by depression (1.32%, 95% CI = 0.91–1.92%), oppositional defiant disorder (1.21%, 95%CI = 0.77–1.87) and attention-deficit hyperactivity disorder (0.84%, 95% CI = 0.52–1.36%). Of the 805 children with a psychiatric disorder, 15.2% had two or more comorbid disorders.

**Conclusions:**

Approximately one in ten Chinese school children has psychiatric disorders involving a level of distress or social impairment likely to warrant treatment. Prevention, early identification and treatment of these disorders are urgently needed and pose a serious challenge in China.

## Introduction

Psychiatric disorders have a significantly adverse impact on children and adolescents, as well as their parents and families, particularly in relation to quality of life. Accurate estimates of the prevalence for these age groups are essential for setting up adequate services and diminish the consequences of mental disorders on later development and functioning in adulthood. Moreover, valid, reliable, not just of symptoms but also of resultant distress and social impairment, screening and diagnostic measures based on multiple informants can contribute to more precise prevalence estimates [1]–[2], such as the Strengths and Difficulties Questionnaire (SDQ) [3] and Development and Well-Being Assessment (DAWBA) [4]. Individual, family and socio economic status (SES) characteristics may play a significant role in the onset of psychopathology in this developmental age. A few studies have shown higher prevalence rates of psychiatric disorders in children and adolescents who live in developing countries when compared to their peers from developed countries, probably due to their poor socioeconomic conditions and the higher environmental difficulties faced by the children and adolescents who live in less developed countries [5]. It is reported that the prevalence rate of psychiatric disorders in children and adolescents from developing countries was between 5 and 18% [6], the wide variation in the prevalence of psychiatric disorders between children suggested that it is important to carry out studies in different cultures.

China is the most populous developing country with 197 million schoolchildren (6–18 years old) in 2011. The family planning policy, large scale rural to urban population transmission and rapidly increasing divorce rate, have produced large amount of “one child”, “left-behind” and “single-parent family” children in China. The highly competitive education system, high expectation from one-child family, and rapidly changing socioeconomic status has made Chinese schoolchildren face increasing stress [7]. In the past decade, many studies have reported the high detection rates of psychiatric problems in Chinese schoolchildren with different instruments, showing big variations from 10.7% to 27.6% [8]–[12]. It's estimated that at least 30 million Chinese children and adolescent under 17 years old are troubled with various emotional or behavior problems [13]. However, only two studies have investigated the total prevalence of psychiatric disorders (not just specific disorders or psychiatric problems) in Chinese children and adolescents in Hunan province using a two-phase design [14]–[15]. A pioneering survey conducted in 1990 reported that 14.89% of 8644 children and adolescents aged 4–16 years had DSM-III-R disorders [14], the second study conducted in 2005 reported 16.22% of 9495 schoolchildren aged 5–17 years had DSM-?? disorders [15]. Both studies have used the screening and diagnostic instruments specifically developed by combing Chinese questionnaire or criteria, such as “Screening Questionnaire for Child Mental Health” [14]–[15] and “Chinese Classification and Diagnosis Criteria of Psychiatric Disorders” [14], with international standardized questionnaire or criteria, such as Kiddie-Schedule for Affective Disorders and Schizophrenia- Present and Lifetime Version (K-SADS-PL) [15]. Therefore, no study to date has used internationally validated instruments, such as SDQ and DAWBA, for child and adolescent psychiatric epidemiology to evaluate the prevalence of psychiatric disorders among the Chinese children and adolescents. Most studies on the mental health in Chinese children/adolescents employed various questionnaires, e.g., Rutter Questionnaire [11], Child Behavior Checklist/Teacher Report Form (CBCL/TRF) [8], [10], Symptom Checklist (SCL-90) [9] or SDQ [12]. Despite their well-established psychometric properties, they are not diagnostic tools and their results do not necessarily map on to formal psychiatric diagnosis. Information on the prevalence of child and adolescent mental health in China is clearly lacking. In this article, we present the first large-scale survey of DSM-IV disorders in China, focusing on prevalence, risk factors, and comorbidity of psychiatric disorders of Chinese schoolchildren.

## Materials and Methods

### Ethics statement

The study was conducted in accordance with the Declaration of Helsinki on ethical principles for medical research involving human subjects. The ethics committee of Liaoning Provincial Center for Disease Control and Prevention approved of the study. Parents were informed about the study and given an information sheet describing the study. Children were included if their parents had given written informed consent. This was considered to be the most appropriate mode as the study was of a simple, observational design with minimal risk.

### Sample

The study was a cross-sectional survey of Chinese schoolchildren aged 6–17 years, attending primary (1st–5th grade), junior (6th–8th grade) and senior schools (9th–11th), and living in three cities (Shenyang, Panjin and Benxi) and three rural counties (Donggang, Zhangwu and Qingyuan) in Liaoning province located in northeast China, which were selected according to areas (urban vs rural) and economic development status. Two–stage sampling was conducted separately at each site by randomly selecting a sample of schools and then by obtaining a random sample of individuals at each selected school. The schools at each site were sampled with stratification by funding source (public/private) and type (banded into key and common schools). The number of schools for each stratum was selected with probabilities proportional in size of the subgroups of students to reflect the public/private ratio, key/common ratio and the neighborhood affluence of the different urban areas. In the first screening phase, the adult version SDQ questionnaire was administered to parents and teachers, and the child version to schoolchildren and adolescent aged 11 and over through the schools in fall 2008.

**Figure 1:**
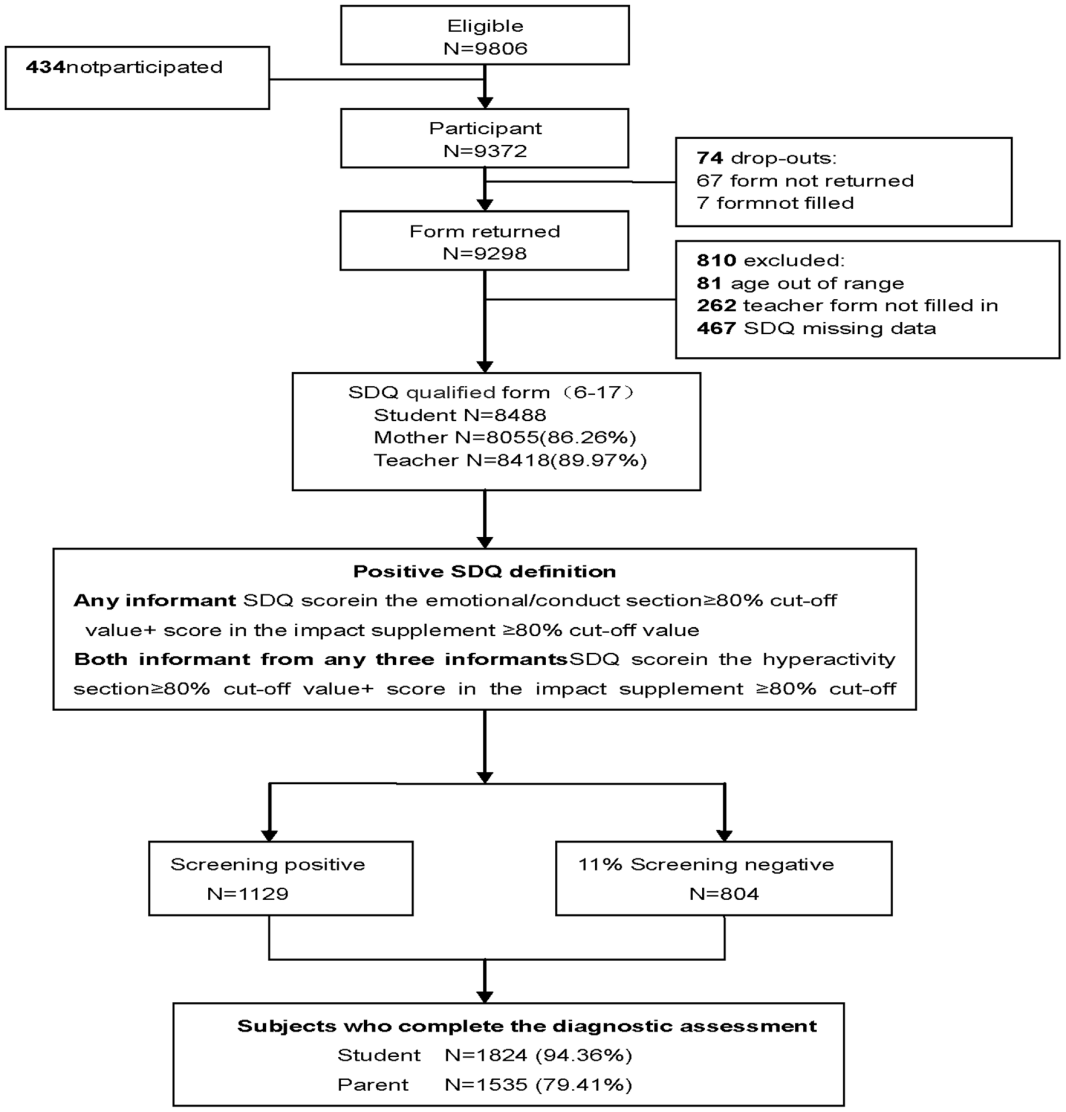
Flowchart showing inclusion criteria for the samples of adolescents in this two-phase design study.

Figure 1 illustrates the subject flow in the two study phases. Of a total of 9806 students eligible for the screening phase, 434 (4.43%) refused to participate, and 74 did not return the questionnaire. Of the 9298 returned forms, 810 (8.71%) were subsequently excluded because the age of 81 was out of the target range (6–17) and 729 with unqualified SDQ. Therefore, information was collected on 8488 students, comprising completed SDQ from 8055 (86.26%) mothers, 5446 (90.57%) schoolchildren aged 11–17 years and 8418 (89.94%) teachers. Children were defined as screen positives if (1) the emotional and/or conduct section scores was above the 80th percentile cutoff and the impact supplement score was above the 80th percentile cutoff (≥2) for anyone of the three informants; (2) the hyperactivity section score was above the 80th percentile cutoff and the impact supplement score was above the 80th percentile cutoff (≥2) for any two of the three informants. Phase 2 was conducted on all screen-positive participants and on an 11% random sample of screen–negative participants. Of 1129 screen-positive and 804 of 7359 screen-negative schoolchildren, DAWBA face to face interviews were completed for 1824 (94.36%) students, 1535 (79.41%) mothers and 1787 (92.45%) teachers. The mean age of the total sample was 11.0 years (SD 2.3 years), and boys made up 53% of the total sample (662 of 1,251).

### Instruments

#### The socio-demographic form

Demographic information on children and adolescent (gender, age, school attended, academic performance, school bullying, academic performance, repeated grades, domestic violence, life events, parenting style, disease history, birth weight, feeding pattern, number of children) and their parents (educational level, marital status, employment, income, general health status, mental disorders, satisfaction with child, smoking, drinking). Left-behind children is defined as children whose father or mother leave hometown and does not live together for over 12 months.

### The Strengths and Difficulties Questionnaire: SDQ

It is a screening questionnaire for mental health problems consisting of 25 items. The items are divided into five subscales with five items each, resulting in scores of emotional symptoms, conduct problems, hyperactive behavior, peer relationships, and prosocial behaviors. All items of the first four scales generate the total difficulties score. An extended version of the questionnaire was used. It contained six extra items assessing the impact of symptoms in terms of suffering, social impairment and burden for relatives, although only five are considered for impact scoring. The Chinese version of SDQ and a computer algorithm, as programmed in SAS version 6.12 (SAS Institute Inc.), is available from the SDQ web site (http://www.sdqinfo.com). The Chinese version of the SDQ has been validated in China [16]. After obtaining written consent forms from parents, an informant-rated version of the SDQ were self-completed by the parents and teachers of the students, while a self-report version of the SDQ was completed by student aged 11–17 year olds themselves, either at school or at home between January and June 2008.

This screening phase allowed children to be divided into “screen positive” and “screen negative” children using by the SDQ algorithm (http://www.iop.kcl.ac.uk/Iop/Departments/ChildPsy/sdq/a1.stm). The original a priori algorithm predicted that a disorder was probably present on the basis that the relevant symptom score was above the 95th centile and the impact score was two or more, which previously has been shown to work well in settings as diverse as Britain and Bangladesh [17]. According to the results of a pilot study, we adjusted such a priori criteria to keep the sensitivity of the screening phase and children were taken to be screen positive if (1) they exceeded the 80th percentile of the emotional and/or conduct section scores and the 80th percentile of the impact supplement score for anyone of the three informants; or (2) they exceeded the 80th percentile of the hyperactivity section score and the 80th percentile of the impact supplement score for any two of the three informants. Phase 2 was conducted on all screen-positive participants and on an 11% random sample of screen–negative participants.

### Development and wellbeing assessment: DAWBA

Clinical assessment was conducted using the parent and adolescent versions of the DAWBA diagnostic interview [4], which uses a mixture of closed and open questions about child psychiatric symptoms and their impact. The version for parent was used for both the mothers and teachers of all children and adolescents, and the version of adolescent was only used to interview adolescent aged 11–17 years. The Chinese version of DAWBA was provided by the interview's author (Robot Goodman), and a previous study has shown satisfactory validity and reliability [18]. The interviews were administrated by trained layman interviewers who also record verbatim accounts of any problems reported, but not rate them. All the diagnoses were made by two experienced child psychiatrists who had completed the online training available for the DAWBA. The raters reviewed preliminary computer-generated diagnoses by computer algorithm (available at http://www/dawba.com) on the basis of symptom and impairment data independently, using information collected from mothers, teachers and schoolchildren, and then the verbatim transcripts in order to accept or modify the computer-generated diagnoses, and assign not-otherwise-specified (NOS) diagnoses. When in doubt, the cases were discussed with a senior child and adolescent psychiatrist until a clear consensus was reached. Interrater agreement between the raters was calculated from a random sample of 100 interviewed participants. The kappa coefficient of 0.75 was within the range as the coefficient yielded by the original English version of the interview [18]. To avoid small cell sizes and wide confidence intervals, the specific diagnoses were categorized into internalizing disorders: separation anxiety, specific phobia, social phobia, panic disorder, post-traumatic stress disorder (PTSD), obsessive-compulsive disorder (OCD), generalized anxiety disorder (GAD) and major depressive disorder (MDD); or externalizing disorders: attention deficit and hyperactivity disorder (ADHD), oppositional defiant disorder (ODD) and conduct disorder (CD). Correlates and regression analyses are presented only for “Any DSM-IV diagnosis”.

### Statistical analysis

We used the SPSS-17 complex sample analysis module (SPSS Inc, Chicago, IL) to calculate the weighted prevalence rates, which uses Taylor series linearization methods to adjust appropriately for sampling weights and clustering within strata in complex surveys designs. The proportions of patients diagnosed in the second phase have been weighted back to allow for the sampling of those screen negatives in the first phase to represent patients originally screened using weights proportional to the inverse of the probability of selection of each subject [19]. Odds ratios (ORs) estimated by logistic regression were used to indicate strength of associations between potential correlates and disorders, as well as the comorbidity between any two of the five major types of disorder (anxiety, depression, ADHD, oppositional defiant, and conduct disorders) while adjusting for the third group.

## Results

Table 1 showed that the weighted prevalence of any DSM-IV psychiatric disorder was 9.49% (8.01%–11.10%), internalizing disorders (7.03%, 5.58%–8.46%) were more frequent than externalizing disorders (2.34%, 1.72%–3.18%). Anxiety disorders showed the highest prevalence rate (6.06%), followed by disruptive disorders (1.63%), depressive disorders (1.32%) and ADHD (0.84%). Special phobia (3.59%), oppositional defiant disorder (1.21%), major depression (1.04%), generalized anxiety disorder (1.09) and obsessive-compulsive disorder (1.00%) were the top five individual disorders. 2.51% of the children had two or more disorders.

**Table 1:** Weighted prevalence (95% CI) for DSM-IV disorder in children aged 6–17.

	Prevalence	95%CI
Any psychatric diagnosis	9.49	8.10–11.10
Internalizing disorder	7.03	5.83–8.46
Anxiety disorders		
Any anxiety disorder	6.06	4.95–7.40
Separation anxiety disorder	0.92	0.57–1.51
Special phobia	3.59	2.70–4.76
Social phobia	0.11	0.10–0.13
PTSD	0.06	0.05–0.08
Obsessive-compulsive disorder	1.00	0.61–1.63
Generalized anxiety disorder	1.09	0.72–1.65
Panic attack	0.34	0.18–0.62
Anxiety NOS	0.91	0.55–1.49
Depressive disorders		
Any depressive disorder	1.32	0.91–1.92
Major depression	1.04	0.71–1.54
Other depressive disorder	0.04	0.03–0.05
Depression NOS	0.39	0.16–0.95
Externalizing disorder	2.34	1.72–3.18
Attention-deficit hyperactivity disorder		
Any ADHD	0.84	0.52–1.36
ADHD combined	0.30	0.27–0.34
ADHD inattentive	0.21	0.08–0.55
ADHD hyperactive	0.37	0.14–0.95
Disruptive disorder		
Any oppositional-conduct disorder	1.63	1.12–2.36
Oppositional defiant disorder	1.21	0.77–1.87
Conduct disorder	0.62	0.39–0.99
Disruptive disorder NOS	0.11	0.02–0.69
Less common disorders	0.97	0.56–1.67
Eating disorders	0.54	0.25–1.14
Tic disorders	0.44	0.20–0.97
Comorbitity		
1	6.87	5.65–8.33
≥2	2.51	1.90–3.31

Note: No subjects were diagnosed with agoraphobia. CI = confidence interval; NOS = not otherwise specified; ADHD = attention-deficit/hyperactivity disorder; less common disorder: tic disorders and eating disorders

Table 2 showed significantly higher prevalence of externalizing (RR = 2.89, 95%CI = 1.45–5.68) in male than in female. Rural children showed non-significantly lower prevalence of externalizing disorders (RR = 0.58, 95%CI = 0.29–1.13) than urban counterparts. Younger children (6–10 years) have significantly lower prevalence of any disorders and internalizing disorders than elders ones, children aged 11–14 years have the highest prevalence of internalizing and internalizing disorders. The prevalence of any and internalizing disorders increase significantly with maternal education levels. Children from divorced or low income families have 2–5 fold increased internalizing and externalizing disorders. Children left-behind had significantly elevated externalizing (RR = 3.48, 95%CI = 1.64–7.37) and any disorders (RR = 1.03, 95%CI = 1.03–2.57), but not internalizing disorders. The prevalence of internalizing and externalizing disorders increased significantly with reducing academic performance records of the children. No significant differences were observed between children from families with one or more children.

**Table 2:** Prevalence of psychiatric disorders by socio-demographic and geographic characteristics.

	Any disorder		Internalizing		Externalizing	
	Ratio(95%CI)	RR (95%CI)	Ratio(95%CI)	RR (95%CI)	Ratio(95%CI)	RR (95%CI)
Gender						
Girl	7.99(6.26–10.15)	1.00	6.69(5.11–8.71)	1.00	1.24(0.71–2.16)	1.00
Boy	10.82(8.75–13.30)	1.40(0.98–1.99)	7.17(5.50–9.29)	1.08(0.72–1.61)	3.48(2.40–5.02)	2.89(1.45–5.68)
District						
Urban	9.16(7.32–11.41)	1.00	6.66(5.08–8.68)	1.00	2.93(2.01–4.25)	1.00
Rural	9.62(7.65–12.03)	1.06(0.74–1.50)	7.21(5.55–9.32)	1.09(0.73–1.63)	1.71(0.99–2.94)	0.58(0.29–1.13)
Age						
6–10	4.33(2.87–6.50)	1.00	2.28(1.20–4.29)	1.00	2.45(1.50–3.98)	1.00
11–14	12.75(10.29–15.70)	3.23(1.97–5.28)	9.80(7.65–12.46)	4.66(2.30–9.45)	2.85(1.81–4.45)	1.17(0.59–2.30)
15–17	10.61(7.92–14.06)	2.62(1.53–4.48)	8.45(6.15–11.49)	3.96(1.90–8.28)	1.34(0.56–3.14)	0.54(0.20–1.47)
Maternal education						
≥9	6.60(4.87–8.88)	1.00	4.54(5.16–6.48)	1.00	1.87(1.07–3.25)	1.00
6–9	9.23(7.20–11.76)	1.44(0.95–2.19)	7.15(5.38–9.46)	1.62(1.00–2.63)	1.58(0.93–2.70)	0.84(0.38–1.85)
0–6	12.99(8.99–18.42)	2.11(1.25–3.57)	9.88(6.46–14.82)	2.30(1.27–4.18)	3.29(1.56–6.83)	1.78(0.69–4.62)
Parental marital status						
Married	8.43(7.08–10.01)	1.00	6.52(5.33–7.95)	1.00	1.65(1.16–2.34)	1.00
Divorced/single	18.16(10.99–28.51)	2.41(1.30–4.46)	12.27(6.60–21.68)	2.01(0.98–4.10)	8.23(3.68–17.41)	5.34(2.12–13.48)
Number of children						
1	8.35(6.85–10.15)	1.00	6.12(4.85–7.72)	1.00	1.86(1.27–2.73)	1.00
≥2	9.14(6.53–12.65)	1.08(0.71–1.65)	7.32(5.02–10.56)	1.21(0.75–1.94)	2.25(1.10–4.56)	1.21(0.53–2.77)
Family income						
High	9.00(6.49–12.34)	1.00	7.07(4.87–10.16)	1.00	1.97(1.00–3.85)	1.00
Middle	6.83(5.38–8.64)	0.74(0.48–1.15)	5.15(3.89–6.79)	0.71(0.44–1.17)	1.39(0.89–2.15)	0.70(0.31–1.59)
Low	19.79(14.31–26.71)	2.50(1.47–4.23)	13.92(9.41–20.12)	2.13(1.17–3.85)	7.10(3.98–12.35)	3.80(1.51–9.54)
Left mother or father						
No	8.19(6.74–9.93)	1.00	6.57(5.27–8.18)	1.00	1.42(0.93–2.15)	1.00
Yes	12.69(8.83–17.90)	1.63(1.03–2.57)	8.11(5.13–12.60)	1.26(0.73–2.16)	4.75(2.62–8.47)	3.48(1.64–7.37)
School record						
High	5.13(3.69–7.07)	1.00	4.12(2.85–5.92)	1.00	0.34(0.30–0.40)	1.00
Middle	11.33(8.73–14.57)	2.37(1.51–3.71)	8.62(6.39–11.53)	2.20(1.33–3.62)	2.33(1.29–4.19)	6.90(3.70–12.88)
Low	16.40(12.73–20.88)	3.63(2.31–5.72)	10.87(7.91–14.76)	2.84(1.69–4.77)	7.14(4.79–10.52)	22.22(14.14–34.92)

### Comorbidity

**Figure 2:**
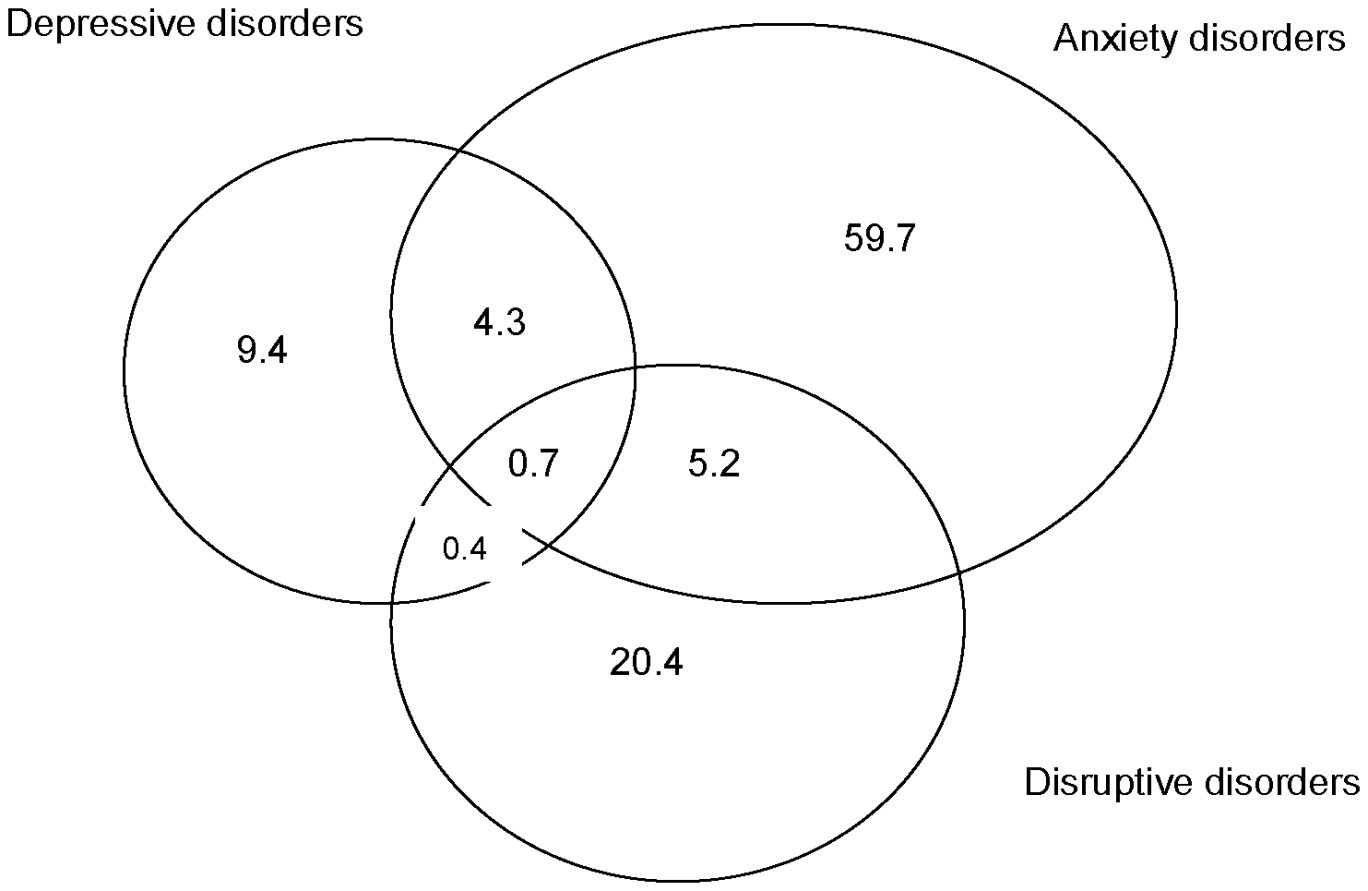
Comorbidity (%) among the 805 children with a psychiatric disorder.

Of the 805 children with any DSM-IV psychiatric disorder, 683 (84.8%) had just one type of disorder (anxiety, depression, ADHD, oppositional-conduct disorder, or less common disorder), 117 (14.5%) had two types of disorder, only 3 (0.4%) and 2 (0.2%) had three and four types. Figure 2 illustrates the comorbidity between anxiety disorders, depressive disorders, and disruptive disorders. Children with depression were most likely to have a comorbid diagnosis (36.4%), followed by children with a disruptive disorder (23.5%), while children with an anxiety disorder have the lowest rate (13.7%).

**Figure 3:**
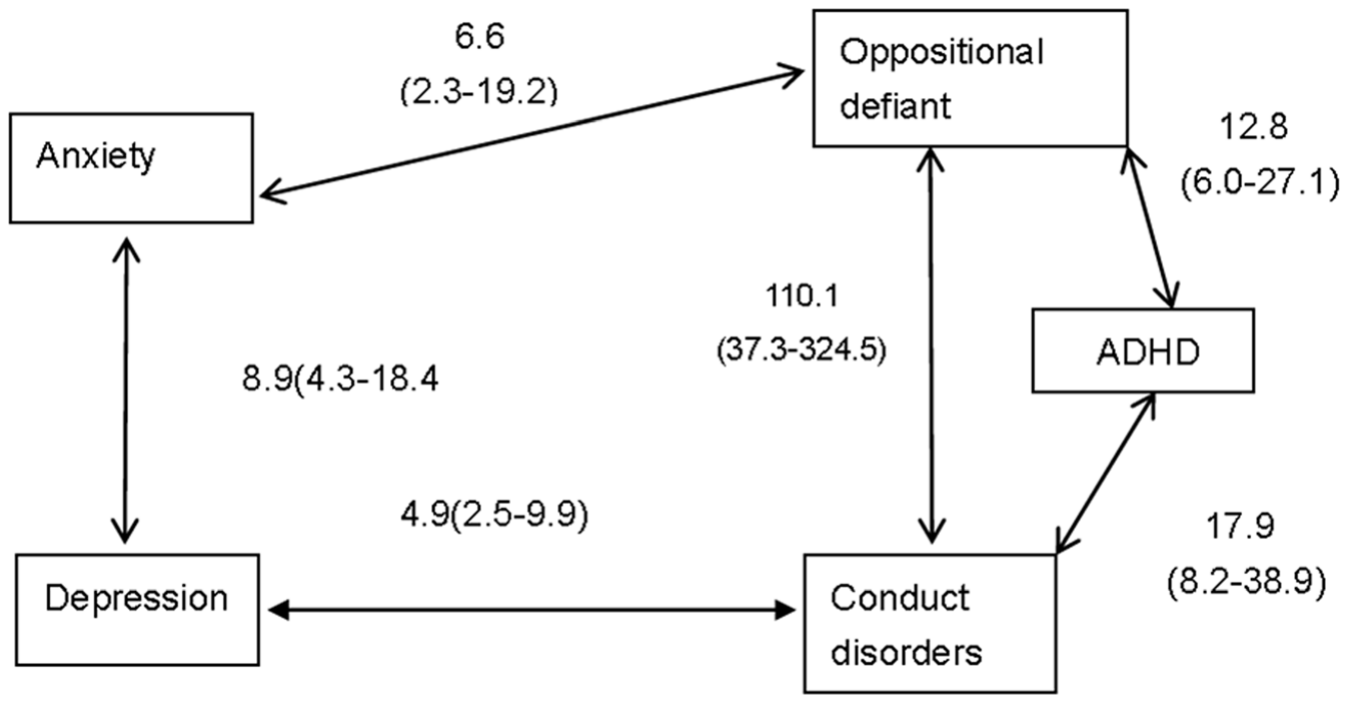
Adjusted odds ratios (95% confidence intervals) for comorbid DSM-IV disorders. ADHD = attention-deficit/hyperactivity disorder.

Figure 3 demonstrates the strength of the adjusted associations between the five main categories of disorder, children with anxiety, depression, ADHD, conduct or oppositional disorder all had significantly increased odds of having another major type of disorder, after adjusting for the presence of other disorders, but there was not a significant association between ADHD and either anxiety or depression, between anxiety and conduct disorder, between depression and oppositional defiant disorder

## Discussion

The total prevalence of 9.49% is almost the same as 9.48% found in the first study with the DAWBA among 5–15 years old British children [20], higher than Indian children aged 12–16 years (1.81%) [21], Norwegian children aged 8–10 years (7.0%) [22] and Italian schoolchildren aged 10–14 years (8.2%) [23], but lower than Brazilian children and adolescent (10.8%–12.7%) [24], Israeli adolescents aged 14–17 (11.7%) [25], Bangladesh children aged 5–10 years (15.2%) [26], Russian children aged 7–14 years(15.3%) [27], and Yemeni schoolchildren aged 7–10 (15.7%) [28]. Although the overall prevalence rate is somehow lower than that found in the low or middle developed countries [26]–[28], it is generally in line with previous studies. The prevalence was also lower than previously reported results in Chinese children and adolescents from Hunan province of mainland China (14.9%–16.2%) [14]–[15], Hong Kong (16.4%) [29] and Taiwan (14.8%–22.7%) [30]. The differences in these studies may reflect the real variations in the prevalence of psychiatric disorders among schoolchildren from different areas in China. Previous studies showed that prevalence rates of mental disorders in children and adolescents may vary greatly, studies using instruments with less restricted criteria for functional impairment have found higher rates [31]–[32]. It's important to note that the range of disorders covered by the DAWBA is somewhat limited compared with the Youth and Parent versions of DISC-IV (Diagnostic and Statistical Manual of Mental Disorders-4^th^ edition) used in Hunan [15], and the Schedule for Affective Disorders and Schizophrenia for School-Age Children–Epidemiologic Version (K-SADS-E) used in Taiwan [30] and Hong Kong [29], and does not specifically address developmental, somatization, psychotic or substance abuse disorders. The differences in the screening and diagnostic instruments, especially the inclusion of a consideration of impairment in both SDQ and DAWBA, may contribute partly to the lower prevalence in the present study.

Although the rates for any mental disorder are similar in Chinese and Great Britain (9.49%:9.48%), anxiety (6.06%:3.77%) and depression (1.32%:0.92%) disorders prevail in the former whereas behavioral disorder (1.63%:3.67%) and ADHD (0.84%:2.23%) prevail in the latter [20]. The pattern of higher prevalence of internalizing disorders and low prevalence of externalizing disorders (7.03% and 2.34%) was similar to the findings in Israeli adolescents (8.1% and 4.8%) [25]. Previous studies have reported that Chinese/Asian children experience more internalizing problems whereas Western children experience more externalizing problems [33]–[37]. It was reported that the rates of total behavior problems in Chinese preschoolers were largely similar to those in American preschoolers, but Chinese preschoolers scored higher on internalizing problems while American preschoolers scored higher on externalizing problems [35]. In the present study, we found similar mean SDQ subscale scores on the conduct, hyperactivity and inattention problems rated by the parent and teacher between Chinese (data not shown) and British children and adolescents [3], but Chinese parents (0.27±0.87 vs 0.37±0.32) and teachers (0.24±0.78 vs 0.30±0.25) rated significantly lower impact score than their British counterparts, implying that Chinese adults tend to undervalue the impact of such problems. Our results supported the previous speculation that Chinese children subjected to socialization practices that stress self-control, emotional restraint, submissiveness, and dependency on others' opinions are more prone to developing internalizing or over-controlled problems [38]–[40], as opposed to externalizing or under-controlled problems [41]–.

Furthermore, the Chinese maybe constitutionally less vulnerable to hyperactivity on the basis of a more placid inborn temperament, some evidence has demonstrated ethnic differences between Chinese and American children in their autonomic nervous systems [43]. Despite previous suggestions of cross-cultural differences between Chinese and Western children or their informants on reporting psychopathology [44], a Chinese ethnicity cannot fully explain here our lower prevalence of ADHD, ODD and CD (0.84%, 1.21% 0.62%, respectively), given the higher rates reported in most previous studies in Chinese children and adolescent (3.3–7.5% for ADHD, 1.3–6.8% for ODD, 1.41–2.9% for CD) [14]–[15], [29]–[30]. Several large-scale DSM-IV population surveys also report more conservative prevalence estimates for the total ADHD prevalence (1.3%–2.5%) [20], [28], [45]–[47].The differences in methodological issues related to age ranges and diagnostic thresholds, especially the consideration of impairment in both SDQ and DAWBA, may contribute partly to the lower prevalence in the present study. Previous studies using the same diagnostic assessment have also showed big differences in the level and pattern of psychiatric disorders by countries or races, emotional and behavioral disorders are commoner than ADHD, but they have not consistently show that emotional disorders are commoner than behavioral disorders or vice versa [6], [18], [20], [24], [27], [45], higher rates of emotional than behavioral disorders were also found in Norway [46], Italy [23], Yemen [28] and Israel [25]. Clearly, the possibility of ADHD being less prevalent than previously believed is important to address in future surveys. We detected only one case of autism spectrum disorder (ASD), the low prevalence could be related to the fact that many children with ASD might stay at home or study in the schools specific for the disabled children, and could not be detected in the school-based survey. Another reason could be that the DAWBA could miss some ASD cases because it focuses on present day symptoms [48].

### Comorbidity

An overall comorbidity rate of 15.2% in the present study is lower than most reports in youth, ranging from 24% to 29% [20], [24], [47], but higher than Yemeni children (11.4%) [28]. In comparison with British children with the similar prevalence of any disorders [20], the pattern of lower ADHD (0.84%) and higher anxiety disorders (6.06%) in Chinese children could contribute to the lower level and different structure of comorbid diagnoses. However, we found almost the same pattern of the associations between disorders as British children, although the ORs were about 1/2 lower, supporting that comorbidity is selective, being evident between anxiety and depression, between ADHD and behavior disorders, and between depression and some behavior disorders [20]. The striking association between ADHD and behavior disorders and the lack of association between ADHD and emotional disorders corresponds to other surveys in which the association with the third group of disorders was properly controlled for in the analysis [20], [24], [47].

### Distribution by democratic factors

We found that externalizing disorders were much commoner among males than females, while internalizing disorders had a relatively even gender balance, both were associated with learning disabilities, divorced family and low SES, this replicates a well-established pattern found in both developed and developing countries [15], [20], [22]–[23], [27]–[28], [31], [45], [47]. Females had insignificantly higher rates of internalizing disorders than males for those aged 6–10 (RR = 1.49, 95%CI = 0.42–5.28) and 15–17 (RR = 1.32, 95%CI = 0.66–2.62), but lower for those aged 11–14 (RR = 0.70, 95%CI = 0.41–1.21). We believe that the absence of gender difference in internalizing disorders could be related to the changing prevalence in school children with different age, similar results have been reported in Yemeni school children [28]. Mental disorders were associated with being elder, rather than younger, for any and internalizing disorders, primarily resulting from the rather lower ADHD in the present study. The higher prevalence of disorder observed for children aged 11–14 could be related to the onset of puberty, especially internalizing disorders [20], [23], [47]. Many studies have suggested that rural children have fewer mental health problems than urban children [26], [28], but the urbanization of previously rural areas can lead to increased mental health problems despite improvements in living conditions, education and physical health [50]. Similar to previous finding in Hunan China [15], the absence of significant urban-rural differences in our study could be the combined effects of complex interactions of multiple factors in a society under rapid urbanization. As expected, the significantly elevated risks of externalizing (RR = 3.48) and total (RR = 1.63) disorders for the left-behind Children (LBC) confirmed previous report that LBC are at higher risk to develop emotional and behavior problems [49]. In the past three decades, millions of Chinese farmers have immigrated to the cities for better life, which is obtained at the expense of family dysfunction and multiple negative impacts on the lives of their children [50]–[51], further attention is required to improve left-behind children's wellbeing, not only the basic daily care and personal safety, but also parental affection and mental health services.

### Limitations

The present results should be viewed in the context of several limitations of the study. First, the survey was conducted among schoolchildren, those children left school because of mental health problem, poor academic achievement or other troubles were not included in the survey. It's therefore conceivable that including all children would have resulted in higher prevalence. Second, the small number of subjects meeting criteria for a diagnosis precluded analysis at the level of individual diagnoses. However, the classification of mental disorders into externalizing and internalizing groups is widely employed and well-validated, and can also contribute to explaining the comorbidity among individual mental disorders. Third, the age and stage of puberty were not assessed, such information could have contributed to elucidate the reasons for the changes in prevalence of mental problems with age. Last, because the teachers have to rate all the students in a class (about 40), they may not have sufficient time to attend to each individual child, it is unclear whether and how this burden may have affected ratings in different sampled areas.

### Conclusions

The total prevalence of psychiatric disorder among Chinese schoolchildren was 9.49%, with higher internalizing disorders than externalizing disorders. There was comorbidity between ADHD and behavior disorders, anxiety and depression, emotional disorders and conduct disorder. Given the total number of 208 million students in all the primary and high schools in China, there would be a minimum of 20 million Chinese children and adolescents have significant mental health problems with serious function damage. Family and SES factors are significantly associated to the development of the psychiatric disorders, more attention should be paid to improve the family function and mental health care for the children, early identification and treatment of these problems are an urgent need and a big challenge for China to develop a national mental health policy for the interests of the children, their families and the society as well. Considering the huge, diverse and geographically spread Chinese population, more studies using internationally validated instruments and methodology from different regions are warranted to contribute to the growth of our knowledge on the mental health of Chinese adolescents.
